# LIPG supports adaption to oxidative stress

**DOI:** 10.17179/excli2019-1555

**Published:** 2019-07-10

**Authors:** Gregor Leonhardt

**Affiliations:** 1 Leibniz Research Centre for Working Environment and Human Factors

## ⁯⁯⁯

***Dear Editor, ***

Recently, Cadenas and colleagues reported that the endothelial lipase (LIPG) is upregulated during oxidative stress and supports survival of cells that are no longer able to generate a sufficient supply of fatty acids by de novo synthesis (Cadenas et al., 2019[3]). LIPG is a cell surface associated lipase that cleaves phosphatidylcholine from high-density lipoproteins (Jaye et al., 1999[10]; Choi et al., 2002[5]). Thereby, free fatty acids are released that can be taken up by cells (Riederer et al., 2012[13]). It has also been shown that overexpression of an oncogenic form of erbB2 leads to strong expression of LIPG (Cadenas et al., 2012[4]) and LIPG has been reported to be associated with tumor growth (Slebe et al., 2016[17]) and with metastasis in triple-negative breast cancer (Lo et al., 2018[11]). 

In the present study, Cadenas and colleagues used overexpression and knockdown strategies to demonstrate that LIPG enables breast cancer cell lines to utilize circulating lipoproteins to synthetize and store triglycerides in lipid droplets (Cadenas et al., 2019[3]). Moreover, the authors showed that oxidative stress under conditions that block endogenous fatty acid synthesis induces LIPG expression and activity. Induction of LIPG was also observed after pharmacological inhibition of de novo fatty acid synthesis (Cadenas et al., 2019[3]). A key observation of the present study is that LIPG upregulation protects the cells from mitochondrial dysfunction and cell death. Finally, analyzing expression data of more than 1,000 breast carcinomas, Cadenas and colleagues showed that a small fraction of tumors overexpresses LIPG which was associated with shorter metastasis-free survival. 

Progression of tumors is a complex process that involves genes controlling proliferation (Schmidt et al., 2008[14]), immune cell infiltration (Schmidt et al., 2012[15]; 2018[16]; Heimes et al., 2017[7][8]; Edlund et al., 2019[6]), redox status (Cadenas et al., 2010[1]), metabolism (Hellwig et al., 2016[9]; Marchan et al., 2017[12]; Stewart et al., 2012[19]) and circadian rhythm (Cadenas et al., 2014[2]) and several more. It is clear that carcinomas have to adapt to conditions of hypoxia and oxidative stress (Spangenberg et al., 2006[18]). LIPG upregulation seems to be one of the mechanisms how cancer cells can guarantee fatty acid supply from extracellular sources under conditions where oxidative stress blocks endogenous synthesis. 
